# Supplementary material for: Immigration Rates during Population Density Reduction in a Coral Reef Fish
Source: PLoS One. 2016 Jun 7;11(6):e0156417. doi: 10.1371/journal.pone.0156417 (PMC4896503; doi:10.1371/journal.pone.0156417)
Supplement: S1 Fig — Location and names of fringing reefs along the west coast of Barbados, West Indies. Reefs are shown as dark green and the area covered by the Barbados Marine Reserve as light blue. On the left, two reefs illustrate the positions of reef zones, with the spur and groove zone used in this study shown in light green. Reproduced with permission of the copyright owner. Source: Turgeon, K. 2011. Home range relocation: How habitat quality, landscape connectivity and density affect movements in coral reef fish. Ph.D thesis. McGill University, Department of Biology. 288 pages. (DOCX) [file pone.0156417.s001.docx]

| 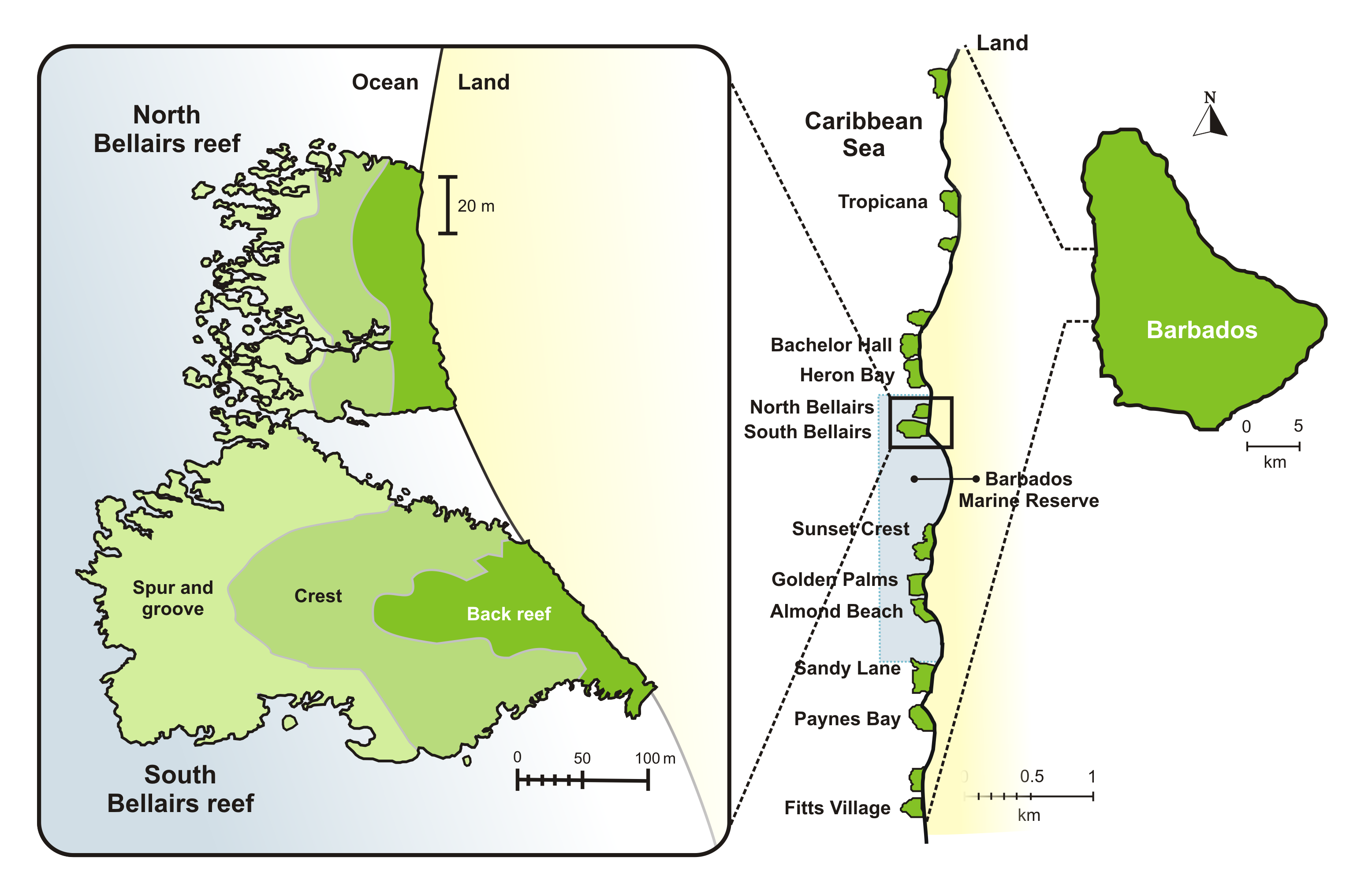 |
| --- |
| **S1 Fig. Location of experimental and control sites**. Location and names of fringing reefs along the west coast of Barbados, West Indies. Reefs are shown as dark green and the area covered by the Barbados Marine Reserve as light blue. On the left, two reefs illustrate the positions of reef zones, with the spur and groove zone used in this study shown in light green. Reproduced with permission of the copyright owner. Source: Turgeon, K. 2011. Home range relocation: How habitat quality, landscape connectivity and density affect movements in coral reef fish. Ph.D thesis. McGill University, Department of Biology. 288 pages. |
