# Supplementary material for: Immigration Rates during Population Density Reduction in a Coral Reef Fish
Source: PLoS One. 2016 Jun 7;11(6):e0156417. doi: 10.1371/journal.pone.0156417 (PMC4896503; doi:10.1371/journal.pone.0156417)
Supplement: S3 Fig — Relationships between the number of damselfish on a site after a removal event and the immigration rate per capita for each individual site and for all sites combined (upper left panel). Individual sites are abbreviated and ordered as in Fig 1. Black lines represent the best fitting empirical function based on maximum likelihood estimates (MLE) of parameter values. For all sites combined, the abscissa indicates the proportion of the initial number of fish on the site before removal. Each point represents the mean ± 1 SE of all points falling within bins of 0.1, with density = 0 shown separately. Sample sizes per bin vary depending on replacement rates. The short inside tick marks on the abscissa represent observed proportion values. The solid lines represent the significant smoothing term functions from gamm models and the dashed lines represent the Bayesian credible intervals. (DOCX) [file pone.0156417.s003.docx]

**S3 Fig. Relationships between immigration and the number of damselfish for *per capita* scaling.** Relationships between the number of damselfish on a site after a removal event and the immigration rate per capita for each individual site and for all sites combined (upper left panel). Individual sites are abbreviated and ordered as in Fig 1. Black lines represent the best fitting empirical function based on maximum likelihood estimates (MLE) of parameter values. For all sites combined, the abscissa indicates the proportion of the initial number of fish on the site before removal. Each point represents the mean ± 1 SE of all points falling within bins of 0.1, with density = 0 shown separately. Sample sizes per bin vary depending on replacement rates. The short inside tick marks on the abscissa represent observed proportion values. The solid lines represent the significant smoothing term functions from gamm models and the dashed lines represent the Bayesian credible intervals.

| 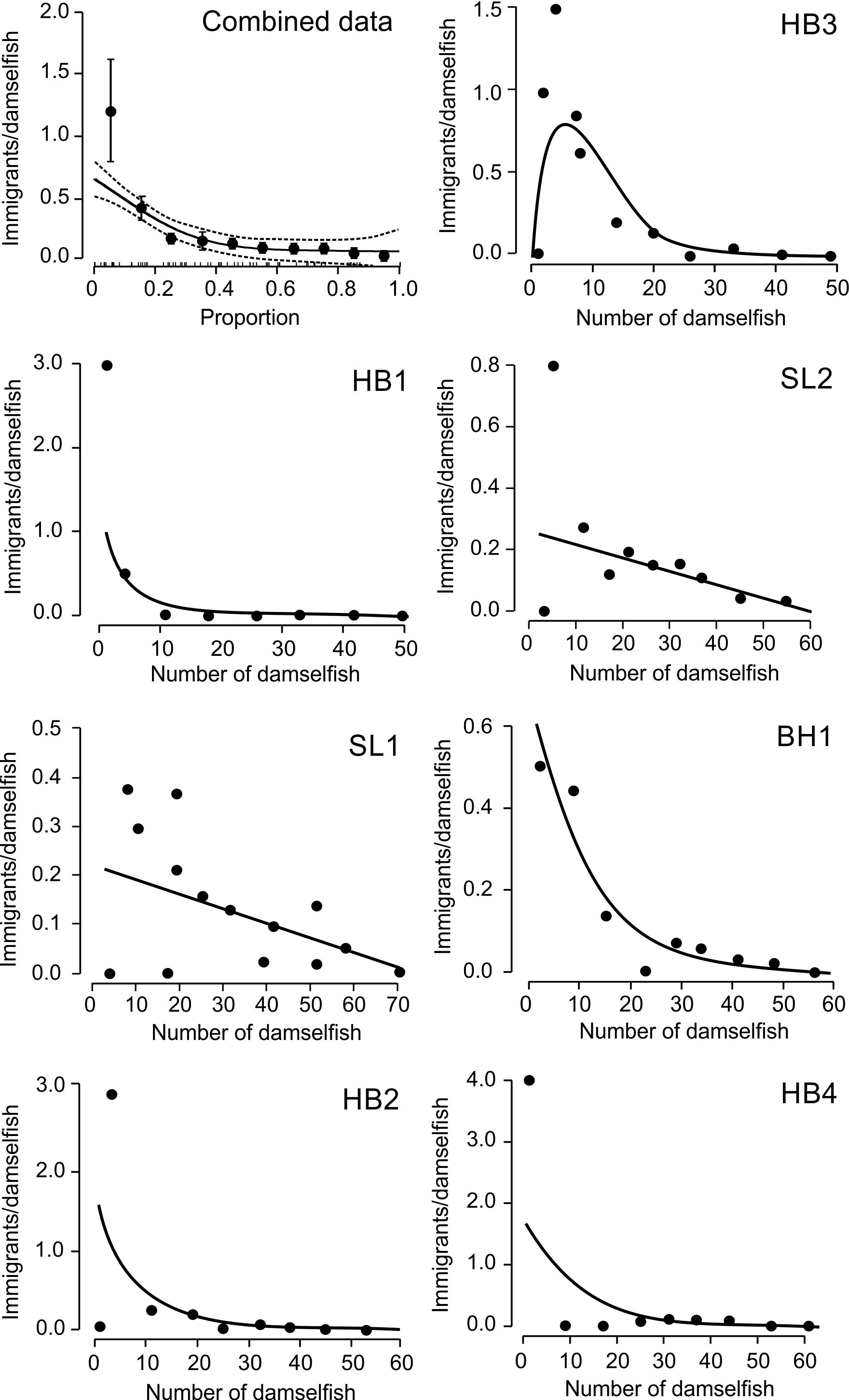 |
| --- |
| S3 Fig |
