# Supplementary material for: Immigration Rates during Population Density Reduction in a Coral Reef Fish
Source: PLoS One. 2016 Jun 7;11(6):e0156417. doi: 10.1371/journal.pone.0156417 (PMC4896503; doi:10.1371/journal.pone.0156417)
Supplement: S1 Table — Habitat variables of seven sites in which the density of two damselfish species (Stegastes diencaeus and S. adustus) was manipulated. See Fig 1 for site abbreviations. (DOCX) [file pone.0156417.s004.docx]

**S1 Table. Habitat variables of the seven sites.** Habitat variables of seven sites in which the density of two damselfish species (*Stegastes diencaeus* and *S. adustus*) was manipulated. See Fig 1 for site abbreviations.

| Habitat variable | Site | | | | | | |
| --- | --- | --- | --- | --- | --- | --- | --- |
|  | HB3 | HB1 | SL2 | SL1 | BH1 | HB2 | HB4 |
| Latitude (13°N) | 11'48.87" | 11'54.93" | 10'17.06" | 10'15.51” | 11'59.27" | 11'51.67" | 11'46.19" |
| Longitude (59°W) | 38'36.84" | 38'36.82" | 38'20.87" | 38'20.85" | 38'36.77" | 38'36.75" | 38'35.93" |
| Depletion area (m^2^) | 157 | 137 | 93 | 147 | 142 | 215 | 142 |
| Hard substrate in depletion area (m^2^) | 72 | 48 | 91 | 83 | 48 | 77 | 110 |
| Number of residents |  |  |  |  |  |  |  |
| *Stegastes diencaeus* | 39 | 34 | 30 | 43 | 33 | 36 | 50 |
| *Stegastes adustus* | 10 | 16 | 34 | 27 | 20 | 17 | 11 |
| Both species combined | 49 | 50 | 64 | 70 | 53 | 53 | 61 |
| Number of potential immigrants |  |  |  |  |  |  |  |
| *Stegastes diencaeus* | 77 | 9 | 155 | 47 | 47 | 18 | 100 |
| *Stegastes adustus* | 37 | 0 | 257 | 94 | 109 | 64 | 20 |
| Both species combined | 114 | 9 | 412 | 141 | 156 | 82 | 120 |
| ^1^ Absolute habitat quality index (AHQ; cm) |  |  |  |  |  |  |  |
| *Stegastes diencaeus* | 10.3 | 7.92 | 8.69 | 8.88 | 8.33 | 7.75 | 9.28 |
| *Stegastes adustus* | 6.73 | 7.03 | 7.66 | 7.39 | 6.87 | 6.82 | 7.60 |

S1 Table. Continued and concluded.

| Habitat variable | Site | | | | | | |
| --- | --- | --- | --- | --- | --- | --- | --- |
|  | HB3 | HB1 | SL2 | SL1 | BH1 | HB2 | HB4 |
| ^2^ Relative habitat quality index (RHQ; cm) |  |  |  |  |  |  |  |
| *Stegastes diencaeus* | 1.072 | -1.073 | 0.181 | 0.648 | -1.430 | 0.672 | -0.716 |
| *Stegastes adustus* | -0.371 | . | -0.141 | 0.094 | -0.487 | 0.093 | 0.333 |
| ^3^ Weighted habitat quality index (WHQ) |  |  |  |  |  |  |  |
| *Stegastes diencaeus* | 1.536 | -1.025 | 0.135 | 0.425 | -0.834 | -0.434 | 0.197 |
| *Stegastes adustus* | -1.116 | . | 1.183 | 0.531 | -0.740 | -0.765 | 1.205 |
| ^4^ Structural connectivity index 1 (hard substrate in source area; m^2^) | 399.1 | 51.67 | 451.7 | 200.5 | 297.8 | 372.9 | 328.3 |
| ^5^ Structural connectivity index 2 (weighted proportional index; WPI) | 0.407 | 0.080 | 0.522 | 0.220 | 0.340 | 0.549 | 0.411 |
| ^6^ Functional connectivity index (weighted proportional index adjusted for barriers to movement; WPI_B_) | 0.152 | 0.004 | 0.416 | 0.155 | 0.239 | 0.300 | 0.445 |

^1^ **Absolute habitat quality index (AHQ)**: We used mean damselfish body size as a surrogate for habitat quality based on evidence that larger individuals often obtain preferred territories in many fishes, including damselfishes [1–4]. AHQ represents the attractiveness of the harvested area and is measured by the mean body size of damselfish in the harvested area for each species separately.

^2^ **Relative habitat quality (RHQ)**: RHQ represents the difference in habitat quality between the harvested and the source area and is measured by the mean body size of damselfish in the harvested area minus the mean body size in the source area for each species separately. HB1 did not have *S. adustus* in the source area.

^3^ **Weighted habitat quality (WHQ)**: WHQ represents the combined effects of absolute and relative habitat quality indices. To calculate WHQ, we z-standardized AHQ and RHQ for each species and built 10 alternative models where the weight of AHQ and RHQ varied in 10% increments [*e.g*., Model 1: WHQ = (0*AHQ) + (1*RHQ), Model 2: WHQ = (0.1*AHQ) + (0.9*RHQ)]. We compared the ten alternative models based on their AICc scores and present the one that best predicted total immigration (see [5] for additional details). In *S. diencaeus*, the weighting that provided the highest AICc scores for WHQ was 0.4* RHQ + 0.6* AHQ and in *S. adustus*, the best WHQ consisted of 0.1 * RHQ + 0.9 * AHQ.

^4^ **Structural connectivity index 1 (hard substrate in source area; m^2^)**: This landscape structural connectivity index (*i.e.* derived from physical attributes of the landscape) assumes that damselfish are less likely to move over open sand than over solid reef [7]. This index consists as the hard substrate cover (m^2^) in the source area.

^5^ **Structural connectivity index 2 (WPI)**: The second structural connectivity index used components of a proximity index called the weighted proportion index (*WPI*) and used by [6]. *WPI* uses grid cells instead of habitat patches, making it suitable for landscapes where discrete habitat patches are not readily defined and assumes that damselfish movement is limited by sand and distance from the harvested area.

^6^ **Functional connectivity index (WPI_B_)**: We developed a functional connectivity index by extending the *WPI* to incorporate the size of sand gaps as barriers to movement based on empirical measurements in *S. diencaeus* [7]. See [5] for additional details about the different indices.

**Literature Cited Only in Supporting Information – S1 Table**

1. Lindström K. The effect of resource holding potential, nest size and information about resource quality on the outcome of intruder-owner conflicts in the sand goby. Behavioral Ecology and Sociobiology. 1992;30: 53–58. doi:10.1007/BF00168594

2. Cheney KL, Côté IM. Habitat choice in adult longfin damselfish: territory characteristics and relocation times. Journal of Experimental Marine Biology and Ecology. 2003;287: 1–12. doi:10.1016/S0022-0981(02)00500-2

3. Markert J, Arnegard M. Size-dependent use of territorial space by a rock-dwelling cichlid fish. Oecologia. 2007;154: 611–621. doi:10.1007/s00442-007-0853-5

4. Turgeon K. Home range relocation: how habitat quality, landscape connectivity and density affect movements in coral reef fish. Ph.D Thesis, McGill University. 2011.

5. Turgeon K, Kramer DL. Compensatory immigration depends on adjacent population size and habitat quality but not on landscape connectivity. Journal of Animal Ecology. 2012;81: 1161–1170. doi:10.1111/j.1365-2656.2012.01990.x

6. Winfree R, Dushoff J, Crone EE, Schultz CB, Budny RV, Williams NM, et al. Testing Simple Indices of Habitat Proximity. The American Naturalist. 2005;165: 707–717. doi:10.1086/an.2005.165.issue-6

7. Turgeon K, Robillard A, Gregoire J, Duclos V, Kramer DL. Functional connectivity from a reef fish perspective: behavioral tactics for moving in a fragmented landscape. Ecology. 2010;91: 3332–3342.
