# Supplementary material for: Immigration Rates during Population Density Reduction in a Coral Reef Fish
Source: PLoS One. 2016 Jun 7;11(6):e0156417. doi: 10.1371/journal.pone.0156417 (PMC4896503; doi:10.1371/journal.pone.0156417)
Supplement: S2 Table — AICc scores (AICc) and Akaike weights (wi) for each of the six empirical functions predicting observed immigration rate per new vacancy (number of immigrants/number of damselfish removed in a removal event) in relation to population size following each removal event (not including saturation because new vacancies = 0) on seven experimental sites. Sites are ordered as in Fig 1. For each site, the function with the highest support is indicated by bold AICc and wi. See Fig 1 for site abbreviations and Table 1 for the functions. (DOCX) [file pone.0156417.s005.docx]

| Functions | K | Sites | | | | | | | | | | | | | |
| --- | --- | --- | --- | --- | --- | --- | --- | --- | --- | --- | --- | --- | --- | --- | --- |
|  |  | HB3 | | HB1 | | SL2 | | SL1 | | BH1 | | HB2 | | HB4 | |
|  |  | AICc | *w_i_* | AICc | *w_i_* | AICc | *w_i_* | AICc | *w_i_* | AICc | *w_i_* | AICc | *w_i_* | AICc | *w_i_* |
| DI | 2 | -9.96 | 0.138 | -45.74 | 0.050 | -6.85 | 0.003 | **-7.74** | **0.471** | -16.01 | 0.054 | **-22.04** | **0.425** | **-22.84** | **0.614** |
| LDD | 3 | -11.55 | 0.305 | -44.80 | 0.031 | -6.34 | 0.002 | -4.64 | 0.100 | -14.80 | 0.030 | -21.44 | 0.315 | -21.35 | 0.292 |
| NDD | 3 | -9.03 | 0.087 | **-50.72** | **0.607** | -3.22 | 0.000 | -4.60 | 0.098 | -11.98 | 0.007 | -18.58 | 0.075 | -19.01 | 0.090 |
| SDD | 3 | -6.55 | 0.025 | -36.52 | 0.001 | -2.40 | 0.000 | -4.42 | 0.090 | -11.62 | 0.006 | -17.24 | 0.039 | -10.57 | 0.001 |
| PDD | 3 | -9.55 | 0.112 | -36.52 | 0.001 | **-18.66** | **0.992** | 5.74 | 0.001 | **-21.54** | **0.866** | -10.16 | 0.001 | 33.01 | 0.000 |
| RDD | 3 | **-11.73** | **0.334** | -49.38 | 0.310 | -6.66 | 0.002 | -6.40 | 0.241 | -15.20 | 0.036 | -19.88 | 0.144 | -12.23 | 0.003 |

**S2 Table. Model support (AICc scores) for per new vacancy scaling.** AICc scores (AICc) and Akaike weights (w*_i_*) for each of the six empirical functions predicting observed immigration rate per new vacancy (number of immigrants/number of damselfish removed in a removal event) in relation to population size following each removal event (not including saturation because new vacancies = 0) on seven experimental sites. Sites are ordered as in Fig 1. For each site, the function with the highest support is indicated by bold AICc and w*_i_*. See Fig 1 for site abbreviations and Table 1 for the functions**.**
