# Supplementary material for: Immigration Rates during Population Density Reduction in a Coral Reef Fish
Source: PLoS One. 2016 Jun 7;11(6):e0156417. doi: 10.1371/journal.pone.0156417 (PMC4896503; doi:10.1371/journal.pone.0156417)
Supplement: S3 Table — AICc scores (AICc) and Akaike weights (wi) for each of the six empirical functions predicting observed per capita immigration rate (number of immigrants/number of remaining damselfish) in relation to population size following each removal event (excluding population size = 0) on seven experimental sites. For each site, the function with the highest support is indicated by bold AICc and wi. Sites are ordered as in Fig 1. See Fig 1 for site abbreviations and Table 1 for the functions. (DOCX) [file pone.0156417.s006.docx]

**S3 Table. Model support (AICc scores) for *per capita* scaling.** AICc scores (AICc) and Akaike weights (w*_i_*) for each of the six empirical functions predicting observed *per capita* immigration rate (number of immigrants/number of remaining damselfish) in relation to population size following each removal event (excluding population size = 0) on seven experimental sites. For each site, the function with the highest support is indicated by bold AICc and w*_i_*. Sites are ordered as in Fig 1. See Fig 1 for site abbreviations and Table 1 for the functions.

| Functions | K | Sites | | | | | | | | | | | | | |
| --- | --- | --- | --- | --- | --- | --- | --- | --- | --- | --- | --- | --- | --- | --- | --- |
|  |  | HB3 | | HB1 | | SL2 | | SL1 | | BH1 | | HB2 | | HB4 | |
|  |  | AICc | *w_i_* | AICc | *w_i_* | AICc | *w_i_* | AICc | *w_i_* | AICc | *w_i_* | AICc | *w_i_* | AICc | *w_i_* |
| DI | 2 | -9.35 | 0.015 | -29.30 | 0.040 | -14.20 | 0.041 | -27.37 | 0.105 | -16.50 | 0.032 | -17.66 | 0.059 | -16.77 | 0.082 |
| LDD | 3 | -11.36 | 0.040 | -27.10 | 0.013 | **-19.73** | **0.655** | **-30.82** | **0.590** | -20.98 | 0.300 | -17.42 | 0.052 | -17.88 | 0.142 |
| NDD | 3 | -15.30 | 0.286 | **-35.52** | **0.844** | -16.17 | 0.110 | -27.32 | 0.102 | **-21.70** | **0.430** | **-22.28** | **0.593** | **-20.54** | **0.538** |
| SDD | 3 | -5.18 | 0.002 | -23.70 | 0.002 | -10.27 | 0.006 | -24.06 | 0.020 | 4.62 | 0.000 | -12.06 | 0.004 | 3.56 | 0.000 |
| PDD | 3 | -3.38 | 0.001 | -21.42 | 0.001 | -5.31 | 0.000 | -3.08 | 0.000 | -5.96 | 0.000 | -8.60 | 0.001 | -8.02 | 0.001 |
| RDD | 3 | **-16.96** | **0.657** | -31.30 | 0.102 | -17.23 | 0.188 | -28.48 | 0.183 | -20.52 | 0.238 | -20.86 | 0.292 | -18.90 | 0.237 |
